# Long‐term treatment with nintedanib in Asian patients with idiopathic pulmonary fibrosis: Results from INPULSIS®‐ON

**DOI:** 10.1111/resp.13647

**Published:** 2019-07-22

**Authors:** Jin Woo Song, Takashi Ogura, Yoshikazu Inoue, Zuojun Xu, Manuel Quaresma, Susanne Stowasser, Wibke Stansen, Bruno Crestani

**Affiliations:** ^1^ Department of Pulmonary and Critical Care Medicine, ASAN Medical Centre University of Ulsan College of Medicine Seoul South Korea; ^2^ Department of Respiratory Medicine Kanagawa Cardiovascular and Respiratory Centre Yokohama Japan; ^3^ Clinical Research Center National Hospital Organization Kinki‐Chuo Chest Medical Centre Osaka Japan; ^4^ Peking Union Medical College Hospital Beijing China; ^5^ Boehringer Ingelheim International GmbH Ingelheim am Rhein Germany; ^6^ Boehringer Ingelheim Pharma GmbH & Co. KG Ingelheim am Rhein Germany; ^7^ Service de Pneumologie A, DHU FIRE APHP, Hôpital Bichat Paris France; ^8^ INSERM Université Paris Diderot Paris France

**Keywords:** clinical trials, interstitial lung disease, lung fibrosis, nintedanib

## Abstract

**Background and objective:**

The efficacy and safety of nintedanib in patients with idiopathic pulmonary fibrosis (IPF) were investigated in the placebo‐controlled INPULSIS® trials. All patients who completed an INPULSIS® trial could receive open‐label nintedanib in the extension trial INPULSIS®‐ON.

**Methods:**

We assessed the long‐term efficacy and safety of nintedanib in patients of Asian race who were treated in INPULSIS®‐ON. Analyses were descriptive.

**Results:**

A total of 215 Asian patients were treated in INPULSIS®‐ON, of whom 121 continued nintedanib in INPULSIS®‐ON and 94 initiated nintedanib in INPULSIS®‐ON having received placebo in an INPULSIS® trial. At baseline of INPULSIS®‐ON, the mean (SD) age of Asian patients was 66.3 (7.5) years, 80.5% were males and mean (SD) forced vital capacity (FVC) was 78.9 (19.3) % predicted. Median total exposure to nintedanib in both INPULSIS® and INPULSIS®‐ON was 42.2 months; maximum exposure was 64.1 months. In INPULSIS®, the annual rate (SE) of decline in FVC over 52 weeks in Asian patients was −124 (20) mL/year in the nintedanib group and −218 (24) mL/year in the placebo group. In INPULSIS®‐ON, the annual rate (SE) of decline in FVC over 192 weeks in Asian patients was −127 (11) mL/year. Diarrhoea was reported in Asian patients at event rates of 58.8 and 82.5 events per 100 patient exposure–years in patients who continued and initiated nintedanib in INPULSIS®‐ON, respectively.

**Conclusion:**

The effect of nintedanib on slowing disease progression in Asian patients with IPF is sustained over the long term. Long‐term treatment with nintedanib has an acceptable safety and tolerability profile.

## Abstract 2

In a subgroup analysis of data from the open‐label extension of the INPULSIS® trials, INPULSIS®‐ON, the effect of nintedanib on slowing disease progression in Asian patients with idiopathic pulmonary fibrosis was sustained over the long term. Long‐term treatment with nintedanib had an acceptable safety and tolerability profile.

## INTRODUCTION

Idiopathic pulmonary fibrosis (IPF) is a progressive fibrosing interstitial lung disease characterized by worsening lung function, dyspnoea and impaired quality of life.^1^, ^2^ The reported incidence of IPF varies depending on the methodology used to identify cases.^3^ In a study in Japan based on a retrospective review of medical records, the annual incidence of IPF was estimated as 2.2 cases per 100 000.^4^ IPF has a very poor prognosis, with a median survival in patients not receiving anti‐fibrotic therapy of approximately 3 years.^4^, ^5^

Nintedanib, an inhibitor of multiple tyrosine kinases,^6^ is an approved treatment for IPF. The efficacy and safety of 52 weeks' treatment with nintedanib 150 mg twice daily (bd) in patients with IPF were demonstrated in the Phase III INPULSIS® trials.^7^ In both trials, nintedanib significantly reduced the annual rate of decline in forced vital capacity (FVC) versus placebo. Based on pooled data from both trials, the annual rate of decline in FVC was −113.6 mL/year in patients treated with nintedanib and −223.5 mL/year in patients treated with placebo (between‐group difference: 109.9 mL/year (95% CI: 75.9, 144.0)). Furthermore, nintedanib was associated with a numerical reduction in the risk of an investigator‐reported acute exacerbation (hazard ratio (HR): 0.64 (95% CI: 0.39, 1.05); *P* = 0.08).^7^ The adverse event profile of nintedanib was characterized mainly by gastrointestinal events, particularly diarrhoea.^7^

Recently, the long‐term efficacy and safety of nintedanib were confirmed in the open‐label extension of the INPULSIS® trials, INPULSIS®‐ON, which showed that the effects of nintedanib on slowing disease progression persisted beyond 4 years, and that the safety and tolerability profile of nintedanib over long‐term use was consistent with that observed in shorter studies.^8^ A total of 215 (29%) patients who participated in INPULSIS®‐ON were Asian in race. Here, we report the results of a subgroup analysis of the efficacy and safety of nintedanib in Asian patients in INPULSIS®‐ON.

## METHODS

### Trial design

The INPULSIS® and INPULSIS®‐ON trials were conducted according to the principles laid down in the Declaration of Helsinki and the International Conference of Harmonisation Harmonised Tripartite Guideline for Good Clinical Practice and were approved by local authorities. An independent ethics committee or institutional review board at each participating centre approved the trial protocols. Patients provided informed consent before entering an INPULSIS® trial and reconfirmed informed consent before entering INPULSIS®‐ON. The trials were registered with http://clinicaltrials.gov (NCT01335464, NCT01335477 and NCT01619085).

In the INPULSIS® trials, patients were randomized 3:2 to receive nintedanib 150 mg bd or placebo for 52 weeks (Fig. S1 in Supplementary Information). Dose reductions to 100 mg bd and treatment interruptions were allowed to manage adverse events. Recommendations for the management of diarrhoea and hepatic enzyme elevations were provided to the investigators.^7^ Patients who completed the 52‐week treatment period and follow‐up visit 4 weeks later in an INPULSIS® trial were eligible to enter INPULSIS®‐ON. Patients who were receiving nintedanib 150 mg bd or its placebo at the end of an INPULSIS® trial received nintedanib 150 mg bd in INPULSIS®‐ON. Patients who were receiving nintedanib 100 mg bd or its placebo at the end of an INPULSIS® trial could begin INPULSIS®‐ON either on the nintedanib 100 or 150 mg bd dose. After unblinding of the INPULSIS® trials, patients were allowed to increase their dose from 100 to 150 mg bd during INPULSIS®‐ON. The first patient was enrolled into INPULSIS®‐ON in July 2012. The database was locked for final analysis in September 2017.

### End points

The following exploratory efficacy end points were analysed in all patients and in Asian patients in INPULSIS®‐ON: annual rate of decline in FVC over 192 weeks, absolute change in FVC from baseline to week 192, time to first acute exacerbation (as defined by Richeldi *et al*.^7^) and mortality. A time point of 192 weeks was chosen for assessing changes in FVC as this was the time point reached by the last patient who was still receiving nintedanib when the trial ended. Data on adverse events (including acute exacerbations and deaths) were collected until 28 days after treatment discontinuation. Asian race was based on the race selected by patients at enrolment into the INPULSIS® trials. Patients were asked to select their race from the following options: White, Asian, Black/African American, American Indian/Alaska Native and Hawaiian/Pacific Islander.

### Statistical analysis

Efficacy and safety analyses were descriptive and conducted in patients who received ≥1 dose of nintedanib in INPULSIS®‐ON. A random coefficient regression model was used to analyse the annual rate of decline in FVC, with age, height and sex as fixed effects, and patient‐specific intercept and time as random effects. The model used all FVC values from baseline to week 192 to calculate the slope of FVC decline. For patients who discontinued nintedanib, an end‐of‐trial visit was conducted plus a follow‐up visit 28 days after treatment discontinuation; after this, no more data were collected for patients who had discontinued. Kaplan–Meier estimates are presented for time to first acute exacerbation. The incidence of acute exacerbations was calculated as the number of patients with ≥1 acute exacerbation/total years at risk × 100. Total years at risk was considered as the time from the start of treatment to the start of the first event (for patients with an event) or the end of the time at risk (for patients without an event) + 1 day.

Adverse events were coded using the Medical Dictionary for Regulatory Activities (MedDRA) version 20.1. Safety topics of interest included major adverse cardiovascular events (MACE), myocardial infarction and bleeding. Incidence rates of adverse events were calculated per 100 patient exposure–years (PEY).

## RESULTS

### Patients

Of the Asian patients who were treated in the INPULSIS® trials, 239 (74.2%) finished the trial without prematurely discontinuing the trial medication. A total of 215 patients in this cohort were treated in INPULSIS®‐ON, of whom 121 continued nintedanib in INPULSIS®‐ON (having received nintedanib in an INPULSIS® trial) and 94 initiated nintedanib in INPULSIS®‐ON (having received placebo in an INPULSIS® trial). Most (93.0%) of the Asian patients treated in INPULSIS®‐ON started INPULSIS®‐ON on the 150 mg bd dose.

Most Asian patients in INPULSIS®‐ON were from Japan (*n* = 84; 39.1%), China (*n* = 68; 31.6%) or Korea (*n* = 38; 17.7%) (Fig. S2 in Supplementary Information). At baseline of INPULSIS®‐ON, this cohort of patients had a mean (SD) age of 66.3 (7.5) years, 80.5% were males and mean (SD) FVC was 78.9 (19.3) % predicted. The characteristics of Asian patients were similar at baseline of INPULSIS® and INPULSIS®‐ON, except that FVC was slightly lower (78.9 vs 81.2% predicted) at the start of INPULSIS®‐ON. Baseline characteristics were similar between patients who continued and initiated nintedanib in INPULSIS®‐ON (Table 1).

**Table 1:** Characteristics of Asian patients at the start of the INPULSIS® and INPULSIS®‐ON trials

	INPULSIS®		INPULSIS®‐ON	
	Nintedanib (*n* = 194)	Placebo (*n* = 128)	Continued nintedanib (*n* = 121)	Initiated nintedanib (*n* = 94)
Age, years, mean (SD)	66.0 (8.1)	66.0 (7.1)	65.9 (7.6)	66.8 (7.3)
Male, *n* (%)	154 (79.4)	105 (82.0)	98 (81.0)	75 (79.8)
Weight, kg, mean (SD)	66.3 (11.0)	66.2 (10.8)	65.3 (11.2)	66.9 (11.5)
Body mass index, kg/m^2^, mean (SD)	25.0 (3.1)	24.6 (3.1)	24.5 (3.1)	24.9 (3.3)
Ex or current smoker, *n* (%)	140 (72.2)	83 (64.8)	87 (71.9)	60 (63.8)
FVC, % predicted, mean (SD)	81.4 (17.6)	80.8 (18.9)	78.4 (17.6)	79.6 (21.3)

Asian patients were defined by self‐reported race.

FVC, forced vital capacity.

Among Asian patients who continued and initiated nintedanib in INPULSIS®‐ON, respectively, 93 (76.9%) and 77 (81.9%) prematurely discontinued nintedanib during the trial (Fig. S3 in Supplementary Information). Of these 170 patients, 40 patients from Japan discontinued from INPULSIS®‐ON between October and November 2015 after commercial drug became available, based on a regulatory requirement. The median duration of exposure to nintedanib in Asian patients treated in INPULSIS®‐ON was 30.1 months; maximum exposure was 52.3 months (Table S1 in Supplementary Information). The median total exposure to nintedanib in Asian patients who received nintedanib in both INPULSIS® and INPULSIS®‐ON was 42.2 months; maximum exposure was 64.1 months.

Of the Asian patients who continued and initiated nintedanib in INPULSIS®‐ON, respectively, 35 (28.9%) and 42 (44.7%) had ≥1 dose reduction, and 40 (33.1%) and 43 (45.7%) had ≥1 treatment interruption (Table S2 in Supplementary Information). Twelve patients and 15 patients who continued and initiated nintedanib, respectively, had ≥1 dose increase from 100 to 150 mg bd. The majority of Asian patients in INPULSIS®‐ON (68.8%) had a dose intensity (the amount of drug administered over the trial divided by the amount that would have been received had 150 mg bd been administered throughout the trial) of >90%.

### Adverse events

Adverse events in Asian patients in INPULSIS® and INPULSIS®‐ON are shown in Tables 2 and 3. As in the overall trial populations, diarrhoea was the most common adverse event reported in Asian patients. In INPULSIS®‐ON, the event rate of diarrhoea in Asian patients was 58.8 and 82.5 events per 100 PEY in those who continued and initiated nintedanib, respectively (Table 3). Seven (3.3%) Asian patients in INPULSIS®‐ON prematurely discontinued nintedanib treatment due to diarrhoea. Serious adverse events were reported in 145 (67.4%) Asian patients in INPULSIS®‐ON.

**Table 2:** Adverse events in Asian patients in the INPULSIS® and INPULSIS®‐ON trials

	INPULSIS®		INPULSIS®‐ON	
	Nintedanib (*n* = 194)	Placebo (*n* = 128)	Continued nintedanib (*n* = 121)	Initiated nintedanib (*n* = 94)
≥1 Adverse event(s)	181 (93.3)	110 (85.9)	119 (98.3)	94 (100.0)
≥1 Severe adverse event(s)†	43 (22.2)	27 (21.1)	63 (52.1)	46 (48.9)
≥1 Serious adverse event(s)‡	65 (33.5)	37 (28.9)	82 (67.8)	63 (67.0)
≥1 Adverse event(s) leading to permanent drug discontinuation§	42 (21.6)	18 (14.1)	48 (39.7)	43 (45.7)
Progression of IPF¶	5 (2.6)	12 (9.4)	13 (10.7)	15 (16.0)
Decreased appetite	6 (3.1)	1 (0.8)	2 (1.7)	1 (1.1)
Diarrhoea	4 (2.1)	0	4 (3.3)	3 (3.2)
Pneumonia	2 (1.0)	1 (0.8)	1 (0.8)	2 (2.1)
Respiratory failure	0	0	4 (3.3)	0
Weight decreased	0	0	3 (2.5)	0
Lung neoplasm malignant	0	1 (0.8)	2 (1.7)	0
Lung infection	0	0	1 (0.8)	3 (3.2)
Dyspnoea	0	0	1 (0.8)	2 (2.1)
FVC decreased	0	0	1 (0.8)	2 (2.1)

Data are *n* (%) of patients.

† An event that was incapacitating or that caused an inability to work or to perform usual activities.

‡ An event that resulted in death, was immediately life‐threatening, resulted in persistent or clinically significant disability or incapacity, required or prolonged hospitalization, was related to a congenital anomaly or birth defect or was deemed serious for any other reason.

§ Adverse events leading to permanent drug discontinuation in >1.5% of patients in any group shown by the preferred term in MedDRA.

¶ Corresponds to MedDRA term ‘IPF’, which included disease worsening and IPF exacerbations.

FVC, forced vital capacity; IPF, idiopathic pulmonary fibrosis; MedDRA, Medical Dictionary for Regulatory Activities.

**Table 3:** Exposure‐adjusted event rates of most frequent adverse events in Asian patients in the INPULSIS® and INPULSIS®‐ON trials

	INPULSIS®				INPULSIS®‐ON			
	Nintedanib (*n* = 194)		Placebo (*n* = 128)		Continued nintedanib (*n* = 121)		Initiated nintedanib (*n* = 94)	
	Events, *n*	Event rate (per 100 PEY)	Events, *n*	Event rate (per 100 PEY)	Events, *n*	Event rate (per 100 PEY)	Events, *n*	Event rate (per 100 PEY)
Diarrhoea	153	88.5	27	21.6	171	58.8	177	82.5
Nasopharyngitis	50	28.9	37	29.6	65	22.3	56	26.1
Nausea	42	24.3	2	1.6	18	6.2	24	11.2
Progression of IPF†	25	14.5	27	21.6	39	13.4	35	16.3
Upper respiratory tract infection	34	19.7	24	19.2	43	14.8	29	13.5
Lung infection	10	5.8	6	4.8	24	8.3	38	17.7
Decreased appetite	30	17.4	12	9.6	18	6.2	32	14.9
Bronchitis	23	13.3	8	6.4	22	7.6	12	5.6
Cough	19	11.0	11	8.8	13	4.5	25	11.7

Adverse events with event rate >10 per 100 PEY in either group shown by the preferred term in MedDRA.

† Corresponds to MedDRA term ‘IPF’, which included disease worsening and IPF exacerbations.

IPF, idiopathic pulmonary fibrosis; MedDRA, Medical Dictionary for Regulatory Activities; PEY, patient exposure–years.

The event rate of MACE was 5.5 and 2.8 events per 100 PEY in Asian patients who continued and initiated nintedanib in INPULSIS®‐ON, respectively (Table S3 in Supplementary Information). The event rate of myocardial infarction was 1.4 and 0.5 events per 100 PEY in these groups, respectively. The event rate of bleeding was 9.6 and 3.3 events per 100 PEY in these groups, respectively. The incidence rate of elevations in alanine aminotransferase (ALT) and/or aspartate aminotransferase (AST) ≥3× upper limit of normal (ULN) was 4.0 per 100 patient–years both in Asian patients who continued and initiated nintedanib in INPULSIS®‐ON (Table S4 in Supplementary Information). No Asian patients in INPULSIS®‐ON had concomitant ALT and/or AST ≥3× ULN and bilirubin ≥2× ULN (Hy's Law).

### Forced vital capacity

**Figure 1:**
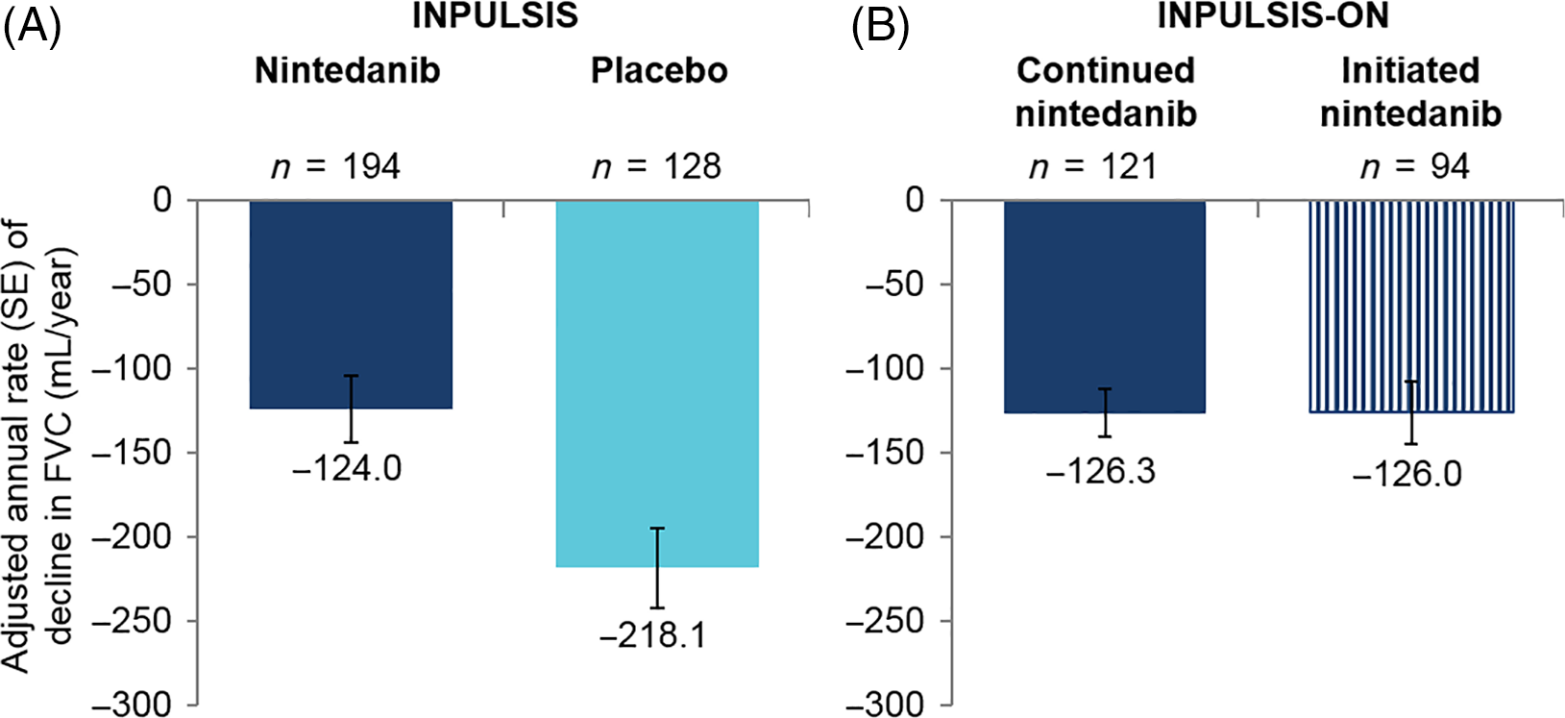
Annual rate of decline in forced vital capacity (FVC) (mL/year) in Asian patients (A) over 52 weeks in the INPULSIS® trials and (B) over 192 weeks (time point reached by the last patient in INPULSIS®‐ON who was still receiving nintedanib when the sponsor stopped the trial) in INPULSIS®‐ON.

**Figure 2:**
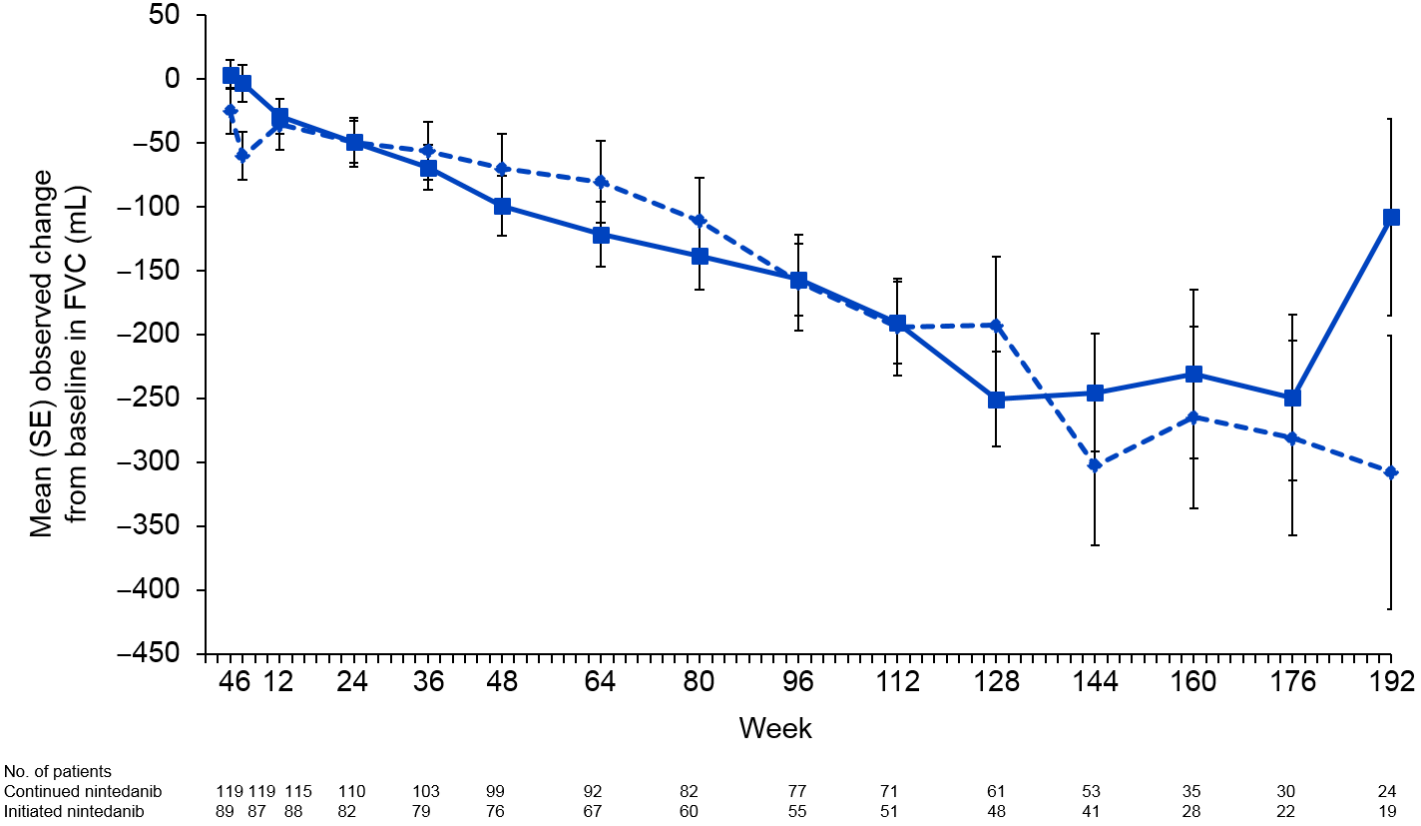
Change from baseline in forced vital capacity (FVC) over time in Asian patients in INPULSIS®‐ON. 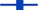, Continued nintedanib; 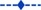, initiated nintedanib.

In INPULSIS®, the annual rate (SE) of decline in FVC over 52 weeks in Asian patients was −124 (20) mL/year in the nintedanib group and −218 (24) mL/year in the placebo group. In INPULSIS®‐ON, the annual rate (SE) of decline in FVC over 192 weeks in Asian patients was −127 (11) mL/year (−126 (14) mL/year in patients who continued nintedanib and −126 (18) mL/year in patients who initiated nintedanib) (Fig. 1). Mean changes from baseline in FVC over time in Asian patients in INPULSIS®‐ON are shown in Figure 2.

### Acute exacerbations and deaths

**Figure 3:**
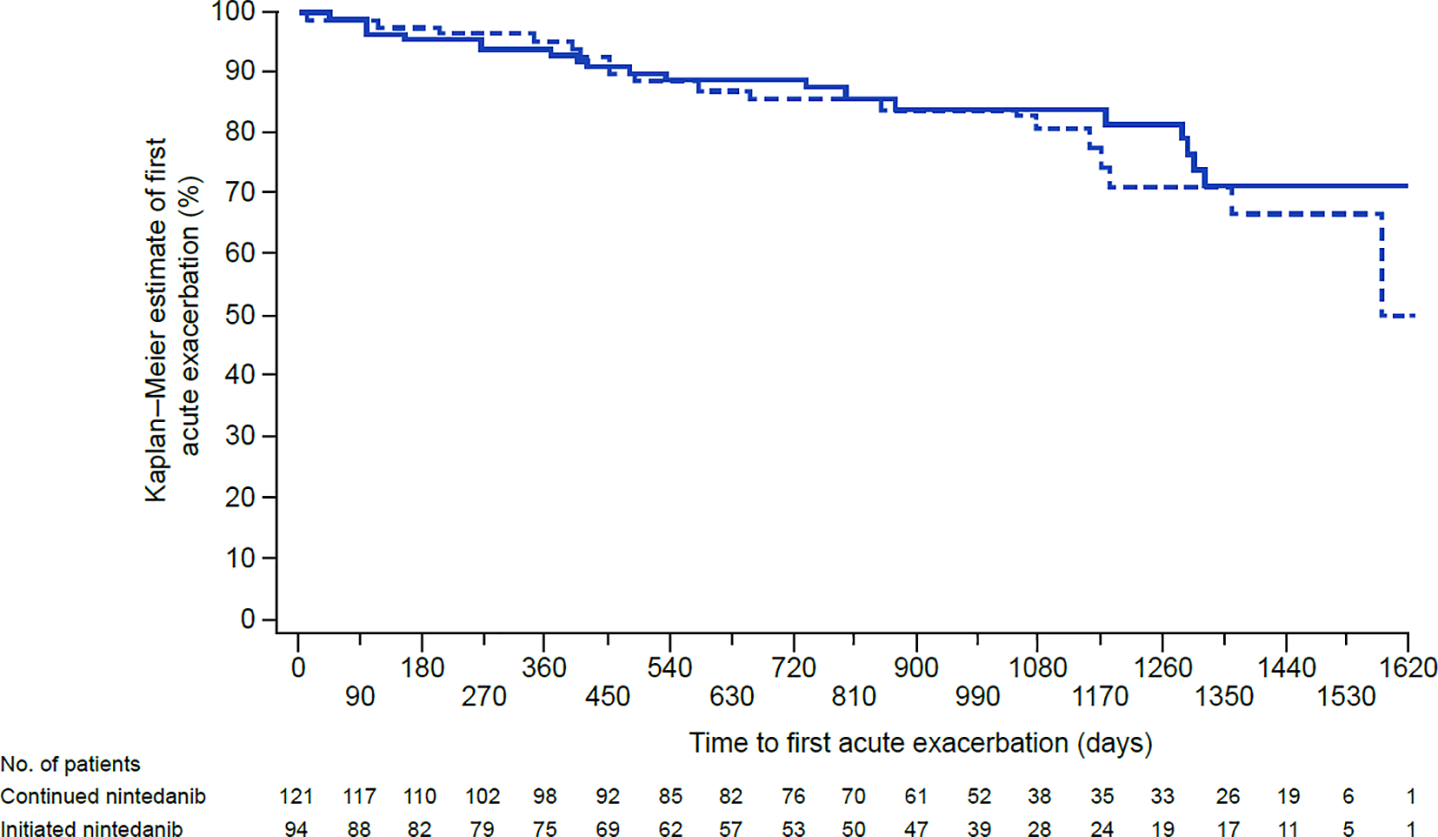
Time to first acute exacerbation in Asian patients in INPULSIS®‐ON. 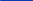, Continued nintedanib; 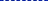, initiated nintedanib.

Among Asian patients in INPULSIS®‐ON, the incidence rate of acute exacerbations was 7.3 per 100 patient–years in patients who continued nintedanib and 8.6 per 100 patient–years in patients who initiated nintedanib (Fig. 3, Table S5 in Supplementary Information). A total of 36 (29.8%) patients who continued nintedanib and 27 (28.7%) patients who initiated nintedanib died over 5 years' follow‐up in INPULSIS®‐ON.

## DISCUSSION

Our analyses of data from the INPULSIS®‐ON trial suggest that the safety and efficacy of nintedanib in Asian patients with IPF are maintained over the long term. These data from Asian patients are consistent with those observed in the overall trial population in INPULSIS®‐ON.^8^ The annual rate (SE) of decline in FVC over 192 weeks in INPULSIS®‐ON was −135 (6) mL/year in the overall population and −127 (11) mL/year in Asian patients. It has been suggested that the course and prognosis of IPF may differ between Asian and White patients.^4^, ^9^, ^10^, ^11^ However, subgroup analyses of data from the INPULSIS® trials showed that the annual rate of FVC decline in placebo‐treated patients, and the effect of nintedanib on FVC decline, was consistent between Asian and White patients^12^, ^13^ and between Japanese patients and the overall population.^14^ In the INPULSIS® trials, the proportion of patients who had an acute exacerbation was similar in the Asian subgroup as in the overall trial population, with Asian race *not* identified as a variable that increased the risk of acute exacerbations in a formal risk factor analysis.^15^ In INPULSIS®‐ON, 39 (18.1%) Asian patients had an acute exacerbation compared with 100 (13.6%) patients in the overall trial population. It is unclear whether the small differences observed in the rates of acute exacerbations between Asian and White patients reflect differences between races or simply different methodologies used to recognize acute exacerbations in different countries. Caution must be exercised in the interpretation of these data, given the small number of events observed and the challenges in classifying events as acute exacerbations of IPF.

The safety and tolerability profile of nintedanib observed in Asian patients participating in the INPULSIS® and INPULSIS®‐ON trials were consistent with that observed in the overall trial populations,^7^, ^8^ with diarrhoea being the most commonly reported adverse event. A similar safety and tolerability profile was recently reported in a real‐world study of Korean patients treated with nintedanib.^16^ In Asian patients in INPULSIS®‐ON, treatment discontinuations due to diarrhoea were uncommon (3% of patients). Data from the overall trial population in INPULSIS®‐ON indicate that discontinuations due to diarrhoea occurred most frequently in the first year of the trial.^17^ Event rates of MACE, myocardial infarction and bleeding in Asian patients in INPULSIS®‐ON were low and consistent with those observed in the overall trial population.^8^

A shortcoming of the present analysis was that patients chose their race from a small list of options provided to them, which resulted in a group of ‘Asian’ patients with various genetic and cultural backgrounds. As with all open‐label studies, interpretation of the data from INPULSIS®‐ON data is limited by the lack of a comparator group. Patients in the INPULSIS® trials whose disease progressed more slowly, or who had fewer/less severe adverse events, would have been more likely to complete the INPULSIS® trial and thus enter INPULSIS®‐ON. In addition, patients with a more benign course of disease might have been more likely to remain on treatment in INPULSIS®‐ON, so potentially reducing the FVC decline and mortality observed in INPULSIS®‐ON. However, as all available FVC measurements collected between baseline and week 192 were used to calculate the rate of decline in FVC, patients with rapid decline in FVC and early discontinuation contributed equally to the calculation as those with slower decline who were still on treatment at week 192.

In summary, results from a subgroup analysis of data from INPULSIS®‐ON, the open‐label extension of the INPULSIS® trials, suggest that the effect of nintedanib on slowing disease progression in Asian patients with IPF is sustained over the long term. Long‐term treatment with nintedanib, up to 64 months, had an acceptable safety and tolerability profile, consistent with that observed in shorter studies.

## Disclosure statement

Some of the data from this study were presented at the annual congress of the Asian Pacific Society of Respirology (APSR) 2018. The INPULSIS® and INPULSIS®‐ON trials were funded by Boehringer Ingelheim. Editorial assistance, supported financially by Boehringer Ingelheim, was provided by Julie Fleming and Wendy Morris of FleishmanHillard Fishburn during the development of this article. The authors were fully responsible for all content and editorial decisions, were involved at all stages of development and provided their approval on the final version. J.W.S. has received personal fees from Boehringer Ingelheim and BridgeBio, and grants from The National Research Foundation of Korea. T.O. has received grants and personal fees from Boehringer Ingelheim; personal fees from Astellas Pharma Inc., Shionogi & Co., Ltd., Toray Industries, Inc., AstraZeneca K.K., AMCO, Inc. and Kyorin, Inc.; and grants from the Japanese Ministry of Health, Labour and Welfare. Y.I. has been an advisor/board member for clinical trials for Boehringer Ingelheim, Shionogi & Co., Ltd. and Toray Industries; has received lecture fees from Boehringer Ingelheim and Shionogi & Co., Ltd.; and has received grants from the Japan Agency for Medical Research and Development, Japanese Ministry of Health, Labour and Welfare and National Hospital Organization in Japan. Z.X. declares no conflicts of interest. B.C. has received grants from Apellis and MedImmune, grants and personal fees from Boehringer Ingelheim and Roche, and personal fees from AstraZeneca and Sanofi. M.Q., S.S. and W.S. are employees of Boehringer Ingelheim.

## Author contributions

Conceptualization: J.W.S., T.O., Y.I., Z.X., B.C., M.Q., S.S., W.S. Formal analysis: W.S. Investigation: J.W.S., T.O., Y.I., Z.X., B.C. Writing—original draft: J.W.S. Writing—review and editing: J.W.S., T.O., Y.I., Z.X., B.C., M.Q., S.S., W.S.

**Abbreviations**
ALT: alanine aminotransferase
AST: aspartate aminotransferase
FVC: forced vital capacity
IPF: idiopathic pulmonary fibrosis
MACE: major adverse cardiovascular event
MedDRA: Medical Dictionary for Regulatory Activities
PEY: patient exposure–year
ULN: upper limit of normal

## Supporting information


**Figure S1** Designs of the INPULSIS® and INPULSIS®‐ON trials.
**Figure S2** Location of Asian patients in the INPULSIS® and INPULSIS®‐ON trials.
**Figure S3** Disposition of Asian patients in the INPULSIS® and INPULSIS®‐ON trials.
**Table S1** Exposure in Asian patients in the INPULSIS® and INPULSIS®‐ON trials.
**Table S2** Dose reductions and treatment interruptions in Asian patients in the INPULSIS®‐ON trial.
**Table S3** Major adverse cardiovascular events, myocardial infarction and bleeding in Asian patients in the INPULSIS® and INPULSIS®‐ON trials.
**Table S4** Hepatic enzyme elevations in Asian patients in the INPULSIS® and INPULSIS®‐ON trials.
**Table S5** Acute exacerbations in Asian patients in the INPULSIS® and INPULSIS®‐ON trials.

## Data Availability

Data are available upon request, which can be submitted via https://trials.boehringer-ingelheim.com/trial_results/clinical_submission_documents.html.
