# Supplementary material for: Long‐term treatment with nintedanib in Asian patients with idiopathic pulmonary fibrosis: Results from INPULSIS®‐ON
Source: Respirology. 2019 Jul 22;25(4):410–6. doi: 10.1111/resp.13647 (PMC7154738; doi:10.1111/resp.13647)
Supplement: Supplementary file 1 — Figure S1 Designs of the INPULSIS® and INPULSIS®‐ON trials. Figure S2 Location of Asian patients in the INPULSIS® and INPULSIS®‐ON trials. Figure S3 Disposition of Asian patients in the INPULSIS® and INPULSIS®‐ON trials. Table S1 Exposure in Asian patients in the INPULSIS® and INPULSIS®‐ON trials. Table S2 Dose reductions and treatment interruptions in Asian patients in the INPULSIS®‐ON trial. Table S3 Major adverse cardiovascular events, myocardial infarction and bleeding in Asian patients in the INPULSIS® and INPULSIS®‐ON trials. Table S4 Hepatic enzyme elevations in Asian patients in the INPULSIS® and INPULSIS®‐ON trials. Table S5 Acute exacerbations in Asian patients in the INPULSIS® and INPULSIS®‐ON trials. [file RESP-25-410-s001.docx]

**SUPPLEMENTARY INFORMATION**

**Long-term treatment with nintedanib in Asian patients with idiopathic pulmonary fibrosis: Results from INPULSIS^®^- ON**

Jin Woo Song, M.D., PhD.^1^ Takashi Ogura, M.D.,^2^ Yoshikazu Inoue, M.D., PhD.,^3^ Zuojun Xu, M.D.,^4^ Manuel Quaresma, Lic.,^5^ Susanne Stowasser, M.D.,^5^ Wibke Stansen, PhD.,^6^ Bruno Crestani, M.D., PhD.^7^

^1^Department of Pulmonary and Critical Care Medicine, ASAN Medical Centre, University of Ulsan College of Medicine, Seoul, South Korea; ^2^Department of Respiratory Medicine, Kanagawa Cardiovascular and Respiratory Centre, Yokohama, Kanagawa, Japan; ^3^Clinical Research Center, National Hospital Organization Kinki-Chuo Chest Medical Centre, Osaka, Japan; ^4^Peking Union Medical College Hospital, Beijing China; ^5^Boehringer Ingelheim International GmbH, Ingelheim am Rhein, Germany; ^6^Boehringer Ingelheim Pharma GmbH & Co. KG, Ingelheim am Rhein, Germany; ^7^APHP, Hôpital Bichat, Service de Pneumologie A, DHU FIRE; INSERM, Unité 1152; Université Paris Diderot, Paris, France

**Figure S1-** Designs of INPULSIS^®^ and INPULSIS^®^-ON


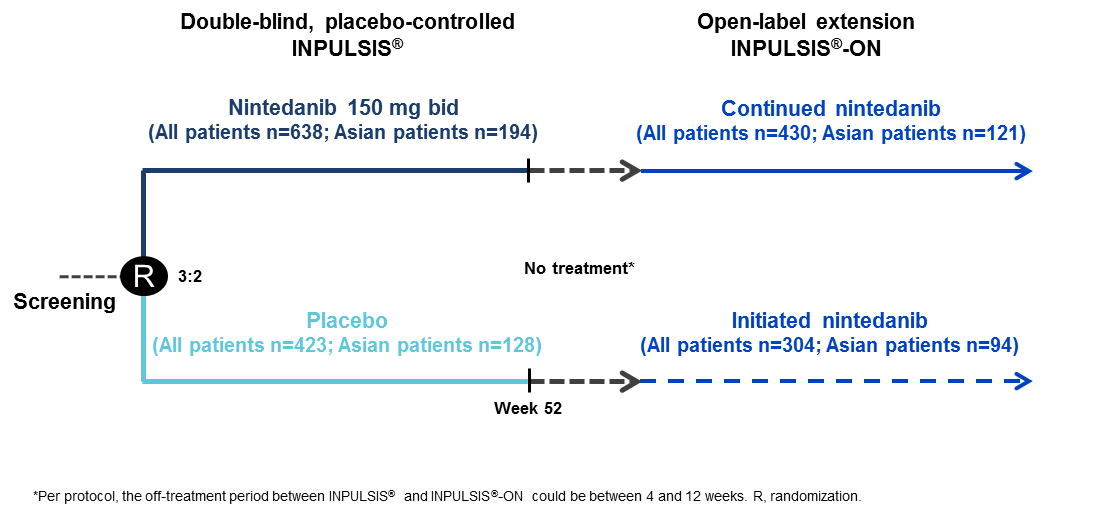


**Figure S2**- Location of Asian patients in INPULSIS^®^ and INPULSIS^®^-ON


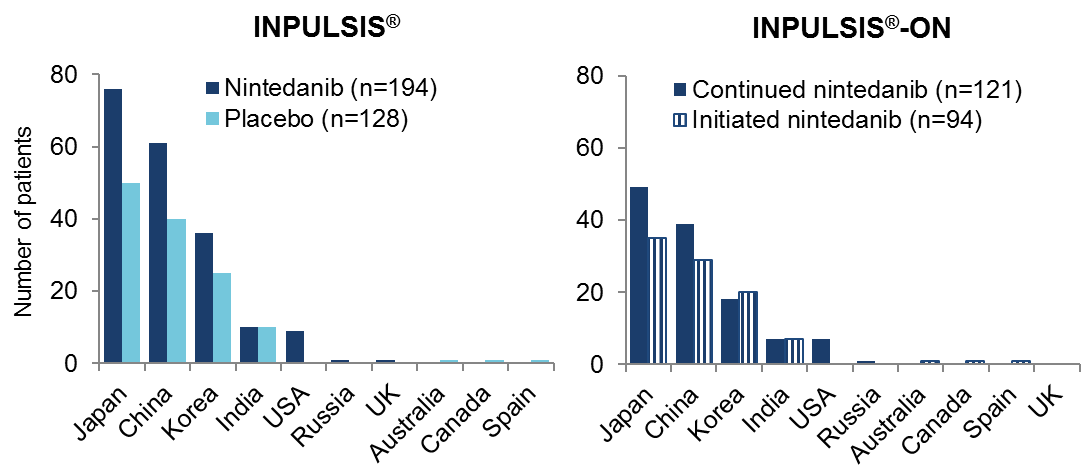


**Figure S3**- Disposition of Asian patients in INPULSIS^®^ and INPULSIS^®^-ON


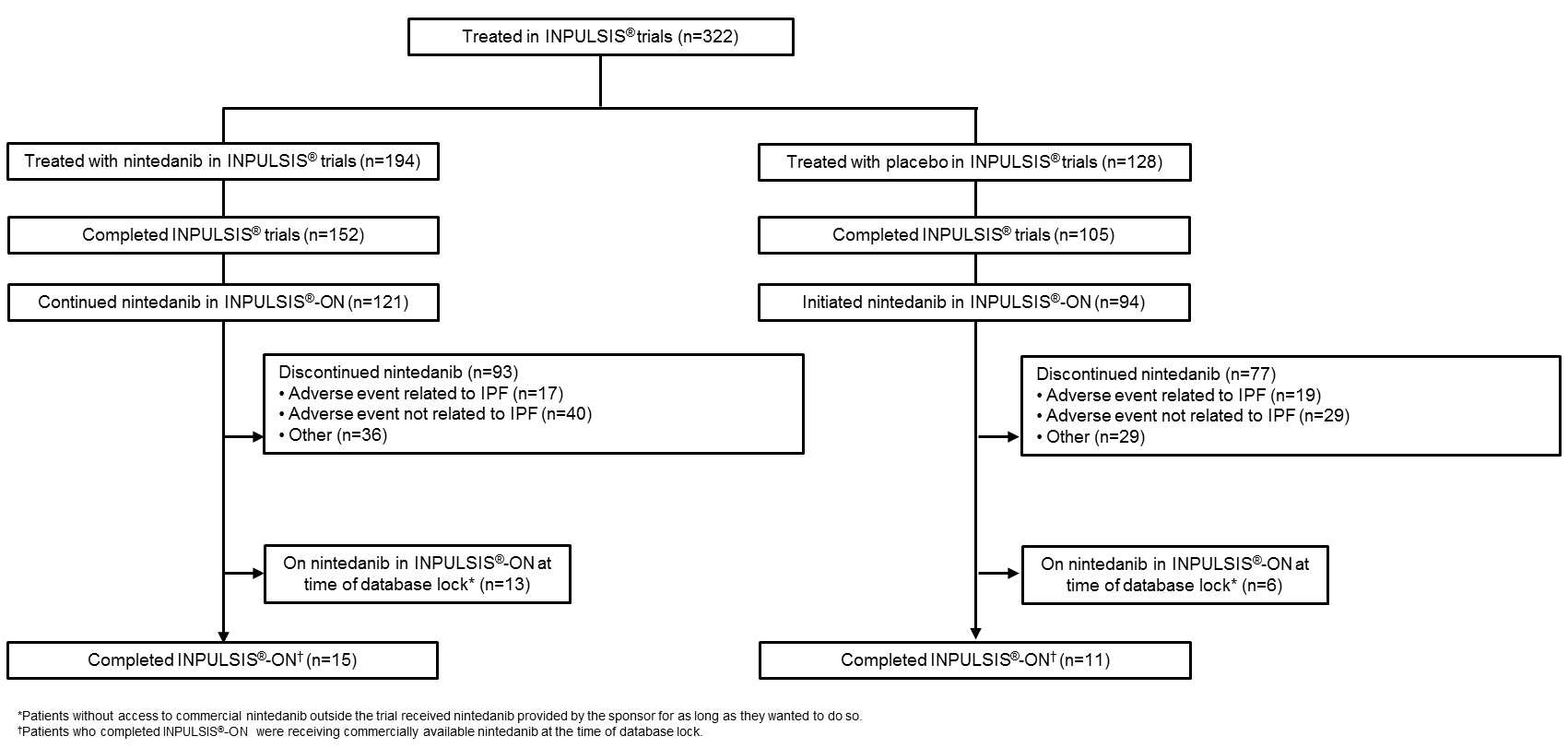


**Table S1-** Exposure in Asian patients in INPULSIS^®^ and INPULSIS^®^-ON

|  | **INPULSIS^®^** | | **INPULSIS^®^-ON** | |
| --- | --- | --- | --- | --- |
|  | **Nintedanib (n=194)** | **Placebo**  **(n=128)** | **Continued nintedanib (n=121)** | **Initiated  nintedanib  (n=94)** |
| Exposure, months |  |  |  |  |
| Mean (SD) | 9.8 (3.8) | 10.8 (2.9) | 28.1 (15.1) | 26.7 (15.8) |
| Median | 11.9 | 11.9 | 30.1 | 30.2 |
| Maximum | 12.4 | 13.1 | 52.2 | 52.3 |
| Total exposure, patient–years | 158 | 116 | 284 | 209 |

**Table S2**-Dose reductions and treatment interruptions in Asian patients in INPULSIS^®^-ON

|  | **INPULSIS^®^-ON** | |
| --- | --- | --- |
|  | **Continued nintedanib**  **(n=121)** | **Initiated nintedanib**  **(n=94)** |
| ≥1 dose reduction to 100 mg bid | 35 (28.9) | 42 (44.7) |
| ≥1 dose increase to 150 mg bid | 12 (34.3)* | 15 (35.7)* |
| Last dose of 150 mg bid | 79 (65.3) | 62 (66.0) |
| ≥1 treatment interruption | 40 (33.1) | 43 (45.7) |
| Re-introduction at 150 mg bid | 14 (35.0)^†^ | 18 (41.9)^†^ |
| Re-introduction at 100 mg bid (after dose reduction) | 14 (35.0)^†^ | 15 (34.9)^†^ |
| Re-introduction at 100 mg bid (same dose as before interruption) | 12 (30.0)^†^ | 10 (23.3)^†^ |

**Table S3-** Major adverse cardiovascular events, myocardial infarction, and bleeding in Asian patients in INPULSIS^®^ and INPULSIS^®^-ON

|  | **INPULSIS^®^** | | | | **INPULSIS^®^-ON** | | | |
| --- | --- | --- | --- | --- | --- | --- | --- | --- |
|  | **Nintedanib**  **(n=194)** | | **Placebo**  **(n=128)** | | **Continued nintedanib**  **(n=121)** | | **Initiated**  **nintedanib**  **(n=94)** | |
|  | **Events, n** | **Event rate (per 100 PEY)** | **Events, n** | **Event rate (per 100 PEY)** | **Events, n** | **Event rate (per 100 PEY)** | **Events, n** | **Event rate (per 100 PEY)** |
| Major adverse cardiovascular events | 6 | 3.5 | 4 | 3.2 | 16 | 5.5 | 6 | 2.8 |
| Myocardial infarction (broad scope) | 4 | 2.3 | 3 | 2.4 | 4 | 1.4 | 1 | 0.5 |
| Myocardial infarction (narrow scope) | 2 | 1.2 | 0 | 0 | 4 | 1.4 | 0 | 0 |
| Bleeding | 20 | 11.6 | 5 | 4.0 | 28 | 9.6 | 7 | 3.3 |

Major adverse cardiovascular events were based on fatal adverse events included in the MedDRA system organ classes “cardiac disorders” and “vascular disorders”; fatal and non-fatal events in the subordinate SMQ “myocardial infarction”; stroke based on selected preferred terms from the subordinate SMQs “haemorrhagic cerebrovascular conditions” and “ischaemic cerebrovascular conditions”; and the MedDRA preferred terms “sudden death”, “cardiac death” and “sudden cardiac death”. Myocardial infarction was based on events in the subordinate SMQ “myocardial infarction”. SMQs include narrow and/or broad terms; narrow terms are those that are highly likely to represent the condition of interest whereas broad terms cover all possible cases, including some that may prove to be of no interest on closer inspection. Bleeding was based on the SMQ “haemorrhage terms (excluding laboratory terms)”. MedDRA, Medical Dictionary for Regulatory Activities; PEY, patient exposure–years; SMQ, standardised MedDRA query.

**Table S4.** Hepatic enzyme elevations in Asian patients in INPULSIS^®^ and INPULSIS^®^-ON

|  | **INPULSIS^®^** | | | | **INPULSIS^®^-ON** | | | |
| --- | --- | --- | --- | --- | --- | --- | --- | --- |
|  | **Nintedanib**  **(n=194)** | | **Placebo**  **(n=128)** | | **Continued nintedanib**  **(n=121)** | | **Initiated nintedanib**  **(n=94)** | |
|  | **n (%)** | **Incidence rate (per 100 patient–years)** | **n (%)** | **Incidence rate (per 100 patient–years)** | **n (%)** | **Incidence rate (per 100 patient–years)** | **n (%)** | **Incidence rate (per 100 patient–years)** |
| Maximum AST and/or ALT |  |  |  |  |  |  |  |  |
| ≥3× ULN | 16 (8.2) | 9.9 | 1 (0.8) | 0.8 | 11 (9.1) | 4.0 | 8 (8.5) | 4.0 |
| ≥5× ULN | 8 (4.1) | 4.7 | 0 | 0 | 6 (5.0) | 2.1 | 0 | 0 |
| ≥8× ULN | 1 (0.5) | 0.6 | 0 | 0 | 2 (1.7) | 0.7 | 0 | 0 |
| Maximum total bilirubin |  |  |  |  |  |  |  |  |
| ≥1.5× ULN | 5 (2.6) | 2.9 | 0 | 0 | 6 (5.0) | 2.1 | 2 (2.1) | 0.9 |
| ≥2× ULN | 2 (1.0) | 1.2 | 0 | 0 | 1 (0.8) | 0.3 | 0 | 0 |
| Maximum alkaline phosphatase |  |  |  |  |  |  |  |  |
| ≥1.5× ULN | 17 (8.8) | 10.5 | 3 (2.3) | 2.4 | 8 (6.6) | 2.8 | 4 (4.3) | 1.9 |
| ≥2× ULN | 8 (4.1) | 4.7 | 1 (0.8) | 0.8 | 2 (1.7) | 0.7 | 2 (2.1) | 0.9 |
| ALT and/or AST ≥3× ULN and bilirubin ≥2× ULN | 0 | 0 | 0 | 0 | 0 | 0 | 0 | 0 |

ALT, alanine aminotransferase; AST, aspartate aminotransferase.

**Table S5.** Acute exacerbations in Asian patients in INPULSIS^®^ and INPULSIS^®^-ON

|  | **INPULSIS^®^** | | **INPULSIS^®^-ON** | |
| --- | --- | --- | --- | --- |
|  | **Nintedanib (n=194)** | **Placebo**  **(n=128)** | **Continued nintedanib (n=121)** | **Initiated nintedanib  (n=94)** |
| Patients with ≥1 acute exacerbation, n (%) | 10 (5.2) | 10 (7.8) | 21 (17.4) | 18 (19.1) |
| Total years at risk | 184 | 130 | 288 | 210 |
| Adjusted incidence rate of acute exacerbations, per 100 patient–years | 5.4 | 7.7 | 7.3 | 8.6 |
